# On Lining Teeth

**Published:** 1856-10

**Authors:** J. F. Wilson


### ARTICLE VI.

### *On Lining Teeth.*

Prof. J. D. White:—*Dear Sir*—I have been favored for some time with you valuable "News Letter," and often find myself much benefited by some of its contents. I have often thought of expressing my sentiments on subjects relative to our profession through your paper; but, feeling a delicacy as to my competency to do any good, I have deferred until now. Possibly what I now propose to offer as an improvement may be an old thing with many of the dentists of the east, but I am satisfied that it is not in general use.

I will here give you, briefly, my mode of backing teeth. I prepare the work in the usual way, and when ready to fit on the backings, I take platina plate, rolled as thin as letter paper, cut out of this enough to cover the face of the tooth, and fit on as with gold; then withdraw the teeth, (after preparing them all in the same way,) draw the points of the rivets together, and file them almost on a level with the lining. I then set them up in plaster and sand separately; after properly dried, I take small scraps of gold plate, enough, guessing at the quantity, to make a heavy backing, and melt them thoroughly on the platina linings; this makes it adhere firmly to the tooth; then, next in order, is to dress it up with a file, leaving a heavy pure gold backing, ready to set back in the plaster and solder to the plate; it requires but little solder to unite the teeth to the plate, and when accurately done requires but little filing to fit it for the lathe. I experimented on several teeth in this way, breaking them after putting up backings, and found the gold to flow under the platina lining and imbed itself into the tooth, making it much more durable and in every respect more neat than the plan usually pursued. I have been putting up work in this way for some months, and from actual experience, can cheerfully recommend it to the profession. If you think this worthy of notice, you will give it a place in the Dental News Letter, and oblige

Yours, respectfully, J. F. Wilson.

